# Patterns of antimicrobial, multidrug and methicillin resistance among *Staphylococcus* spp. isolated from canine specimens submitted to a diagnostic laboratory in Tennessee, USA: a descriptive study

**DOI:** 10.1186/s12917-022-03185-9

**Published:** 2022-03-08

**Authors:** Jennifer Lord, Nick Millis, Rebekah Duckett Jones, Brian Johnson, Stephen A. Kania, Agricola Odoi

**Affiliations:** Department of Biomedical and Diagnostic Sciences, College of Veterinary Medicine, The University of Tennessee, Knoxville, TN USA

**Keywords:** Antimicrobial resistance, Multidrug resistance, Methicillin resistance, *Staphylococcus*, MRSA, Epidemiology, Dogs, Canine, Tennessee, United States

## Abstract

**Background:**

Multidrug- and methicillin-resistant staphylococci are both veterinary and public health concerns due to their zoonotic potential. Therefore, the objective of this study was to investigate patterns of antimicrobial, multidrug, and methicillin resistance among four *Staphylococcus* spp. commonly isolated from canine clinical specimens submitted to the Clinical Bacteriology Laboratory at the University of Tennessee College of Veterinary Medicine (UTCVM).

**Methods:**

Results of antimicrobial susceptibility testing and *mecA* polymerase chain reaction (PCR) for isolates of four common *Staphylococcus* spp. isolates were obtained from the Bacteriology Laboratory at the UTCVM between 01/01/2006 and 12/31/2017. Cochran-Armitage trend test was used to assess temporal trends of antimicrobial resistance (AMR), multidrug resistance (MDR), and methicillin resistance. Kappa test of agreement was used to assess agreement between the results of PCR and disk diffusion tests.

**Results:**

Most of the 7805 isolates were *S. pseudintermedius* (6453 isolates), followed by *S. coagulans* (860), *S. aureus* (330), and *S. schleiferi* (162)*.* Among *S. pseudintermedius* isolates, 45.5% were MDR, and 30.8% were methicillin-resistant (MRSP). There was a significant temporal increase in MRSP (*p* = 0.017). Chloramphenicol resistance increased among both MRSP and methicillin-susceptible (MSSP) isolates (*p* <  0.0001). Among *S. aureus* isolates, 40.9% were MDR, 37.4% were methicillin-resistant (MRSA), and the proportion of MRSA isolates increased significantly (*p* = 0.0480) over time. There was an increasing temporal trend in the proportion of MDR isolates among MSSP (*p* = 0.0022), but a decrease among MRSP (*p* <  0.0001) and MRSA (*p* = 0.0298). *S. schleiferi* had the highest percentage (56.9%) of methicillin-resistant isolates. Oxacillin disk diffusion was superior to cefoxitin for the detection of *mecA*-mediated resistance and had almost perfect agreement with *mecA* PCR assay for *S. pseudintermedius* (95.4% agreement, kappa (κ) = 0.904; *p* <  0.0001), *S. coagulans* (95.6%, κ = 0.913; *p* <  0.0001) and *S. schleiferi* (97.7%, κ = 0.945; *p* <  0.0001). However, cefoxitin disk diffusion was superior to oxacillin disk diffusion and had almost perfect agreement with mecA PCR assay for *S. aureus* (95.3%, κ = 0.834; *p* <  0.0001).

**Conclusions:**

The levels of resistance and increasing temporal trends are concerning. These findings have implications for treatment decisions and public health due to the zoonotic potential of staphylococci. Continued surveillance and use of antibiograms to guide clinical decisions will be critical.

## Background

*Staphylococcus* bacteria are frequently implicated in opportunistic infections [1, 2]. *Staphylococcus pseudintermedius* is a commensal of canine skin and mucosa, and is commonly implicated in canine otitis and pyoderma [3–6]. It has also been identified as the causative agent of numerous other infections, including wound and surgical site infections, urinary tract infections, and osteomyelitis [7–10]. Carriage of *Staphylococcus aureus,* an important commensal of humans, has been documented in many domestic animals, and *S. aureus * may cause opportunistic infections in these species [11, 12]. Indeed, *S. aureus* has been isolated from various canine infections, although much less frequently in comparison to *S. pseudintermedius* [6, 7, 11, 13, 14]. *Staphylococcus coagulans* (formerly *S. schleiferi* subsp. *coagulans*) and coagulase-negative *S. schleiferi* (formerly *S. schleiferi* subsp*. schleiferi*) have been isolated from healthy dogs and those with inflammatory lesions alike, and these organisms have also been associated with recurrent pyoderma [15–19]*.*

Numerous studies have reported that most canine *Staphylococcus* spp. isolates display resistance to at least one antimicrobial [6, 14, 20, 21]. Varying levels of multidrug resistance (MDR), defined as resistance to at least one drug in three or more classes of antimicrobials [22, 23], have also been detected among staphylococci isolated from companion animal specimens [14, 20, 21, 24, 25]. Moreover, methicillin resistance, which has received major attention in human medicine as the spread of hospital- and community-associated methicillin-resistant *S. aureus* (MRSA) challenges infection control efforts worldwide, is also increasingly being detected among canine *Staphylococcus* isolates, particularly *S. pseudintermedius* [26–31].

The occurrence of MDR and methicillin resistance among canine *Staphylococcus* isolates has important clinical implications. Importantly, infections with resistant isolates may require treatment with antimicrobials considered high priority for human health [32, 33]. Methicillin resistant staphylococci (MRS) express the *mecA* gene or its homolog, *mecC*, and produce an altered penicillin binding protein (PBP), which confers resistance to all β-lactam antibiotics, severely limiting treatment options [31, 34–36]. Therapeutic failures resulting from infections with resistant isolates also present serious clinical challenges to clinicians and increase the financial burdens for animal owners [37, 38]. Furthermore, evidence suggesting cross-transmission of resistant pathogens between animals and humans implies that the expansion of these organisms is also a public health concern [39–42]. *S. pseudintermedius* has been identified in human clinical infections, and previous reports suggest that pet owners are at risk of zoonotic transmission [43, 44]. In addition, while *S. aureus* is mainly a pathogen of humans, pets exposed to methicillin resistant *S. aureus* (MRSA) infections by their owners may play a role in perpetuating MRSA colonization in the household [45]. The potential for exposure to antimicrobial or multidrug resistant *Staphylococcus* isolates among those that are immune-compromised is of particular concern [46].

The existence of AMR, MDR, and methicillin resistance among canine *Staphylococcus* isolates, coupled with the public health implications of zoonotic transmission of these organisms, highlights the importance of monitoring antimicrobial susceptibility patterns. Temporal changes in antimicrobial resistance patterns among canine staphylococcal isolates imply that continued surveillance is essential to identify patterns, trends, and newly emerging resistance [14, 47]. Geographically relevant and timely epidemiologic monitoring is useful for the development of evidence-based antimicrobial use guidelines to inform veterinarians of current best practices. It is also important that surveillance data regarding regional AMR patterns for patients from a broad range of clinical settings, rather than specialty practices alone, are available to practitioners to enable informed antimicrobial selection when empirical therapy is necessary. Therefore, the objective of this study was to investigate patterns of antimicrobial, multidrug, and methicillin resistance among four clinically important *Staphylococcus* species *(S. pseudintermedius, S. aureus, S. coagulans*, and *S. schleiferi*) isolated from canine specimens submitted to the Clinical Bacteriology and Mycology Laboratory at the University of Tennessee College of Veterinary Medicine by both referring veterinarians and teaching hospital personnel between 2006 and 2017.

## Methodology

### Study design and data source

This descriptive retrospective study used laboratory records of canine clinical specimens processed at the Clinical Bacteriology Laboratory at the University of Tennessee College of Veterinary Medicine (UTCVM) between January 1, 2006 and December 31, 2017. Records for a total of 7810 canine clinical specimens positive for *S. pseudintermedius, S. aureus, S. coagulans,* or *S. schleiferi* were available for analysis. The data were assessed for duplicate entries and five were identified and removed, leaving 7805 unique isolates for subsequent analyses. The following data were extracted from the laboratory records: patient identification, medical record number, specimen collection site, *Staphylococcus* species isolated, results of antimicrobial susceptibility tests and *mecA* polymerase chain reaction (PCR) assay. Hospital patient records for the study period were also obtained from the UTCVM veterinary teaching hospital and matched to the laboratory records. The following data were extracted from the hospital records: medical record number, patient type (hospital patient specimen vs. submission by referring veterinarian), age, sex, species, and breed.

### Bacterial isolation, antimicrobial susceptibility testing, and PCR assay

Bacterial isolation and antimicrobial susceptibility tests were performed according to standard practice using previously described methods [47]. Briefly, *Staphylococcus* spp. isolates were identified with a biochemical identification process, using the following tests: tube coagulase, phenol red broth with lactose and with trehalose, and the Voges-Proskauer test [48]. The diagnostic laboratory followed Clinical and Laboratory Standards Institute (CLSI) standards for antibiotic susceptibility testing [49–53]. Antimicrobial susceptibility tests for the majority of bacterial isolates were performed using the disk diffusion method. However, some were tested using a microbroth dilution method using an automated susceptibility testing system [47, 54]. Antimicrobial agents from the following classes were included in the standard antimicrobial susceptibility panel for *Staphylococcus* spp.: β-lactams (ampicillin, amoxicillin/clavulanic acid, cefoxitin, cefpodoxime, cephalothin, oxacillin and penicillin), fluoroquinolones (marbofloxacin), folate inhibitors (trimethoprim/sulfamethoxazole [TMS]), lincosamides (clindamycin), macrolides (erythromycin), phenicols (chloramphenicol), and tetracyclines (tetracycline). Reporting of susceptibility to β-lactam antibiotics in the present study was limited to ampicillin, oxacillin, and cefoxitin. Ampicillin (rather than penicillin) was used to represent susceptibility to penicillinase-labile penicillins in this study because the results of ampicillin testing were available for a larger number of *Staphylococcus* spp. isolates. For isolates deemed to be resistant to multiple drug classes, susceptibility tests for the following agents were performed in addition to the standard panel: amikacin, doxycycline, minocycline, and rifampin. Amikacin susceptibility tests were also performed for all cutaneous isolates, while gentamicin was used for otic and urinary isolates.

Oxacillin zone sizes of ≤17 mm (resistant) and ≥ 18 mm (susceptible) were used as breakpoints for classification of *S. pseudintermedius, S. coagulans*, and *S. schleiferi* [55, 56]. In addition, interpretive criteria recommended for coagulase-negative *Staphylococcus* spp. for cefoxitin were followed for *S. pseudintermedius, S. coagulans,* and *S. schleiferi* [49–53]. Otherwise, the laboratory followed CLSI standards in place during the year of specimen submission, and isolates were classified as susceptible, intermediate, or resistant based on these criteria [49–53]. During 2006, a real-time PCR assay was used for the detection of the *mecA* gene for all *Staphylococcus* spp. isolates, with a cycle threshold value of ≤30 being considered positive [47, 57]. During all remaining years of the study period, conventional PCR was employed for this purpose, using previously described methods [55]. After 2006, *mecA* PCR was performed for all staphylococcal isolates resistant to oxacillin or cefoxitin, as well as for 100 oxacillin-susceptible *S. pseudintermedius* isolates annually.

### Data management

Data management and statistical analysis were performed using SAS Version 9.4 [58]. Medical record numbers were used to join patient data extracted from hospital records to the laboratory records. Patient age was categorized into the following groups: < 3 years of age, 3–6 years, 6–9 years, 9–12 years, and ≥ 12 years. Patient sex categories included: intact male, castrated male, intact female, and spayed female. Patient breed was placed into one of the following categories based on American Kennel Club (AKC) designations: herding, hound, toy, non-sporting, sporting, terrier, and working breed groups [59]. Patients with multiple breeds were coded as “mixed breed,” and those with a listed breed not recognized by the AKC were coded as “other”. Specimen collection sites were categorized as ear, skin, urine or bladder, joint or bone, mucosa, and “other”.

Results of antimicrobial susceptibility tests were excluded from analysis if differing interpretations were obtained upon repeated testing. Thus, results for the following agents were excluded from analysis: amikacin (2 isolates), oxacillin (6 isolates), cefoxitin (2 isolates), and cefpodoxime (2 isolates). Results of *mecA* PCR assay were excluded from analysis for 14 isolates for which repeated test results had differing interpretations. Therefore, results of *mecA* PCR assay were included for a total of 4152 isolates. All antimicrobial susceptibility test results were re-classified into two groups, susceptible and resistant, with the latter encompassing all non-susceptible isolates (those classified as “non-susceptible,” “intermediate,” or “resistant”) [23].

The antimicrobial agents used for susceptibility testing were categorized into the appropriate drug classes. Isolates that were non-susceptible to at least one agent in one or more antimicrobial classes, excluding intrinsic resistance, were classified as AMR [22, 23]. Isolates that were non-susceptible to one or more agents in 3 or more antimicrobial classes, excluding intrinsic resistance, were classified as MDR [22, 23]. Isolates were classified as methicillin-resistant based upon results of phenotypic susceptibility testing, confirmed with positive PCR results for the *mecA* gene.

### Statistical analysis

The Kolmogorov-Smirnov test was used to assess for normality of distribution of patient age, which was found to be non-normally distributed and hence median and interquartile range (IQR) were computed. The following categorical variables were assessed: age group, patient breed category, patient sex, patient type, and specimen collection site. The Cochran-Armitage trend test was used to assess for significant temporal trends in AMR, MDR, and methicillin resistance, using a two-sided *p-*value with a cutoff of ≤0.05. This analysis was performed for *S. pseudintermedius, S. aureus, S. coagulans,* and *S. schleiferi* isolates. In addition, separate analyses were conducted for methicillin-resistant and methicillin-susceptible *S. pseudintermedius* (MRSP and MSSP) and *S. aureus* (MRSA and MSSA) isolates. To facilitate comparison between methicillin-resistant and methicillin-susceptible isolates, temporal trends in antimicrobial and multidrug resistance were displayed in graphs, which were generated using R version 4.1.1 [60]. Chi-square tests (or Fisher’s exact tests where appropriate due to sample size) were used to assess for significant differences in levels of antimicrobial and multidrug resistance between methicillin-resistant and methicillin-susceptible isolates. Kappa tests of agreement were used to assess agreement between the results of *mecA* PCR assay and oxacillin as well as cefoxitin susceptibility tests.

## Results

### Summary statistics

A total of 7805 canine clinical specimens with single isolates that met the inclusion criteria for the study were assessed. The most common species was *S. pseudintermedius*, with 6453 isolates, followed by *S. coagulans* (860 isolates), *S. aureus* (330 isolates), and *S. schleiferi* (162 isolates).

Most *Staphylococcus* spp. isolates included in the study were from the skin (56.2%), followed by ear (16.8%), urine or bladder (13.3%), joint or bone (4.5%), and mucosa (1.6%) (Table 1). Patient age ranged from 2 days to 18 years, with a median of 7 years and IQR of 4 to 10 years. The largest percentage of isolates were from spayed females (46%), followed by neutered males (38.5%), intact males (10.5%), and intact females (5.1%). Sporting breeds had the highest proportion (20.2%) of isolates, followed by mixed breeds (19.3%), toy (15.2%), working (11.1%), non-sporting (9.5%), terrier (9.2%), herding (8.4%), and hound breeds (6.9%). The majority of *Staphylococcus* spp. isolates were obtained from specimens submitted by referring veterinarians (59.1%), while 40.9% were from hospital patients.

**Table 1:** Distribution of *Staphylococcus* spp. isolated from canine specimens submitted to a veterinary diagnostic laboratory in Tennessee, USA (2006–2017)

Variable	Category	Number	Percentage	***p***-value*
**Specimen Site**				< 0.0001
	Ear	1310/7804	16.8	
	Joint/bone	354/7804	4.5	
	Mucosa	122/7804	1.6	
	Skin	4387/7804	56.2	
	Urine/bladder	1041/7804	13.3	
	Other	590/7804	7.6	
**Age**				< 0.0001
	< 3 years	639/4785	13.4	
	3 ⎯ < 6 years	1046/4785	21.9	
	6 ⎯ < 9 years	1309/4785	27.4	
	9 ⎯ < 12 years	1171/4785	24.5	
	≥ 12 years	620/4785	13.0	
**Sex**				0.0098
	Female	305/5989	5.1	
	Female spayed	2752/5989	46.0	
	Male	629/5989	10.5	
	Male castrated	2303/5989	38.5	
**Breed**				< 0.0001
	Herding	492/5864	8.4	
	Hound	404/5864	6.9	
	Mixed	1129/5864	19.3	
	Non-sporting	559/5864	9.5	
	Sporting	1186/5864	20.2	
	Terrier	540/5864	9.2	
	Toy	890/5864	15.2	
	Working	649/5864	11.1	
	Other	15/5864	0.3	
**Patient Type**				< 0.0001
	Clinical	3149/7705	40.9	
	Referral	4556/7705	59.1	

*p-value is for Chi-square test

### Antimicrobial and multidrug resistance patterns

- *S. pseudintermedius*

The majority of *S. pseudintermedius* isolates were resistant to ampicillin (84.3%) (Table 2). Substantial proportions of *S. pseudintermedius* isolates exhibited resistance to gentamicin (27.0%), TMS (44.4%), clindamycin (39.1%), erythromycin (38.8%), and tetracycline (47.9%), while overall resistance to chloramphenicol was low (5.4%). Approximately one-third were methicillin-resistant (30.8%), and 45.5% were MDR. There was a statistically significant (*p* = 0.0170) increasing temporal trend in the proportion of methicillin-resistant isolates. Among the subsets of isolates tested for susceptibility to amikacin (cutaneous and MDR isolates) and rifampin (MDR isolates), few exhibited resistance to amikacin (20/2558) or rifampin (10/1026), and no significant temporal trends were observed for these agents.

**Table 2:** Distribution of antimicrobial and multidrug resistance among *S. pseudintermedius* isolated from canine specimens submitted to a veterinary diagnostic laboratory in Tennessee, USA (2006–2017)

	2006–2008	2009–2011	2012–2014	2015–2017	Total
**Aminoglycosides**					
Gentamicin	19.3 (242/1256)	31.7 (482/1523)	29.2 (297/1016)	27.4 (241/881)	27.0 (1262/4676)***
**β-lactams**					
Ampicillin	84.7 (1020/1204)	83.9 (1277/1523)	84.5 (1566/1854)	84.2 (1499/1781)	84.3 (5362/6362)
Cefoxitin	21.7 (272/1251)	19.9 (301/1510)	19.0 (353/1855)	22.4 (396/1768)	20.7 (1322/6384)
Oxacillin	32.6 (411/1259)	36.9 (559/1514)	34.3 (638/1861)	34.8 (622/1790)	34.7 (2230/6424)
**Fluoroquinolones**					
Marbofloxacin	31 (385/1244)	30.1 (453/1504)	28.3 (523/1848)	29.1 (412/1417)	29.5 (1773/6013)
**Folate inhibitors**					
TMS^a^	42.1 (529/1258)	42.7 (649/1521)	42.5 (791/1863)	49.5 (894/1805)	44.4 (2863/6447)***
**Lincosamides**					
Clindamycin	38.9 (489/1256)	40.3 (613/1522)	39.1 (727/1859)	38.2 (688/1801)	39.1 (2517/6438)
**Macrolides**					
Erythromycin	38.4 (483/1257)	39.7 (604/1522)	39.1 (726/1858)	38.0 (685/1802)	38.8 (2498/6439)
**Phenicols**					
Chloramphenicol	2.5 (31/1258)	3.3 (50/1523)	5.8 (107/1862)	8.7 (157/1805)	5.4 (345/6448)***
**Tetracyclines**					
Tetracycline	52.4 (659/1257)	50.1 (762/1521)	45.9 (855/1862)	44.8 (797/1780)	47.9 (3073/6420)***
**AMR**^b^	87.1 (1096/1259)	86.8 (1322/1523)	86.5 (1611/1863)	86.7 (1567/1808)	86.7 (5596/6453)
**MDR**^c^	44.2 (557/1259)	45.8 (697/1523)	45.1 (840/1863)	46.4 (839/1808)	45.5 (2933/6453)
**MRSP**^d^	26.1 (300/1148)	32.5 (479/1473)	31.7 (585/1844)	31.5 (544/1730)	30.8 (1908/6195)*

^a^Trimethoprim/sulfamethoxazole

^b^Antimicrobial resistance

^c^Multidrug resistance

^d^Methicillin-resistant *S. pseudintermedius*, based on oxacillin disk diffusion testing confirmed with positive *mecA* PCR

*p <  0.05, **p <  0.01, ***p < 0.0001 for Cochran-Armitage trend test

**Fig. 1:**
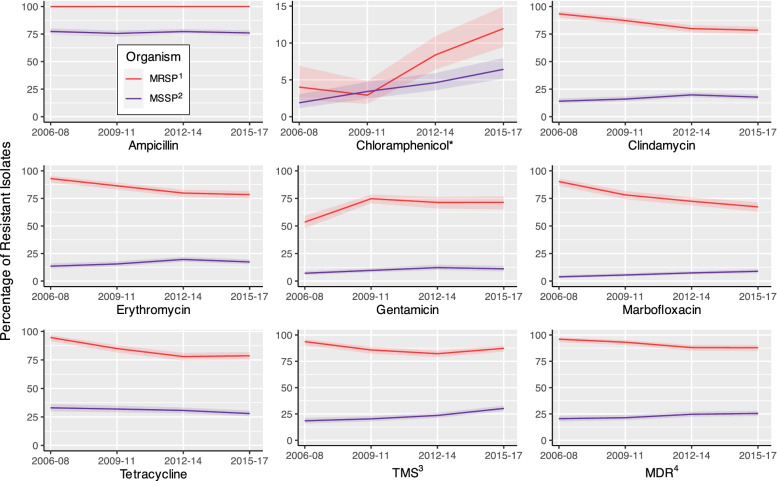
Percentage and 95% confidence intervals of antimicrobial and multidrug resistance among methicillin-resistant and methicillin-susceptible *S. pseudintermedius* isolated from canine specimens submitted to a veterinary diagnostic laboratory in Tennessee, USA (2006–2017). ^1^Methicillin-resistant *S. pseudintermedius*, ^2^Methicillin-susceptible *S. pseudintermedius*, ^3^Trimethoprim/sulfamethoxazole, ^4^Multidrug resistance. *Indicates that upper limit of y-axis has been reduced to improve visual comparison

The MRSP isolates consistently had higher overall levels of antimicrobial and multidrug resistance than the MSSP isolates (Fig. 1). Statistically significant (*p* <  0.0001) differences were noted for ampicillin, chloramphenicol, clindamycin, erythromycin, gentamicin, marbofloxacin, TMS, and MDR. Agents with significant temporal increases among both MRSP and MSSP included gentamicin (*p*_MRSP_ = 0.0001, *p*_MSSP_ = 0.0019) and chloramphenicol (*p* <  0.0001), while tetracycline resistance decreased for both MRSP and MSSP (*p*_MRSP_ <  0.0001, *p*_MSSP_ = 0.0111). In contrast, MDR decreased significantly among MRSP isolates (*p* <  0.0001) but increased among MSSP isolates (*p* = 0.0022), and a similar pattern was observed for several individual antimicrobial agents: marbofloxacin (*p* <  0.0001), TMS (*p*_MRSP_ = 0.0212, *p*_MSSP_ <  0.0001), clindamycin (*p*_MRSP_ <  0.0001, *p*_MSSP_ = 0.007), and erythromycin (*p*_MRSP_ <  0.0001, *p*_MSSP_ = 0.0042).

b) *S. aureus*

Resistance to ampicillin was observed among the majority (82.6%) of *S. aureus* isolates (Table 3). Low levels of resistance were observed for TMS (4.2%), chloramphenicol (2.4%), gentamicin (1.1%), and tetracycline (6.4%), while resistance to clindamycin and erythromycin were relatively high (35.4 and 47.3%, respectively). The overall percentage of MRSA isolates was 37.4%, and increased significantly during the study period (*p* = 0.0480), from 21.6 to 40%. In addition, 40.9% of *S. aureus* isolates were MDR. Among the subsets of isolates tested for susceptibility to amikacin and rifampin, several exhibited resistance to amikacin (12/78), but none were resistant to rifampin (0/21). No significant temporal trends were observed for these agents.

**Table 3:** Distribution of antimicrobial and multidrug resistance among *S. aureus* isolated from canine specimens submitted to a veterinary diagnostic laboratory in Tennessee, USA (2006–2017)

Antimicrobials	2006–2008	2009–2011	2012–2014	2015–2017	Total
**Aminoglycosides**					
Gentamicin	2.5 (2/79)	1.2 (1/86)	0 (0/63)	0 (0/40)	1.1 (3/268)
**β-lactams**					
Ampicillin	83.6 (61/73)	91.9 (79/86)	74.2 (69/93)	81.4 (57/70)	82.6 (266/322)
Cefoxitin	25.6 (20/78)	49.4 (42/85)	42.4 (39/92)	45.7 (32/70)	40.9 (133/325)*
Oxacillin	27.5 (22/80)	52.3 (45/86)	40.2 (37/92)	40 (28/70)	40.2 (132/328)
**Fluoroquinolones**					
Marbofloxacin	23.4 (18/77)	43.5 (37/85)	28.6 (26/91)	40.7 (22/54)	33.6 (103/307)
**Folate inhibitors**					
TMS^a^	3.8 (3/80)	3.5 (3/86)	2.2 (2/93)	8.5 (6/71)	4.2 (14/330)
**Lincosamides**					
Clindamycin	33.8 (27/80)	44.2 (38/86)	33.3 (31/93)	29.0 (20/69)	35.4 (116/328)
**Macrolides**					
Erythromycin	33.8 (27/80)	55.8 (48/86)	48.4 (45/93)	50.7 (36/71)	47.3 (156/330)
**Phenicols**					
Chloramphenicol	3.8 (3/80)	2.3 (2/86)	2.2 (2/93)	1.4 (1/71)	2.4 (8/330)
**Tetracyclines**					
Tetracycline	5.0 (4/80)	7.0 (6/86)	5.4 (5/93)	8.7 (6/69)	6.4 (21/328)
**AMR**^b^	93.8 (75/80)	95.4 (82/86)	89.3 (83/93)	93.0 (66/71)	92.7 (306/330)
**MDR**^c^	35.0 (28/80)	53.5 (46/86)	35.5 (33/93)	39.4 (28/71)	40.9 (135/330)
**MRSA**^d^	21.6 (16/74)	45.8 (38/83)	40.7 (37/91)	40.0 (26/65)	37.4 (117/313)*

^a^Trimethoprim/sulfamethoxazole

^b^Antimicrobial resistance

^c^Multidrug resistance

^d^Methicillin-resistant *S. aureus*, based on cefoxitin disk diffusion testing confirmed with positive *mecA* PCR

*p < 0.05, **p < 0.01, ***p < 0.0001 for Cochran-Armitage trend test

**Fig. 2:**
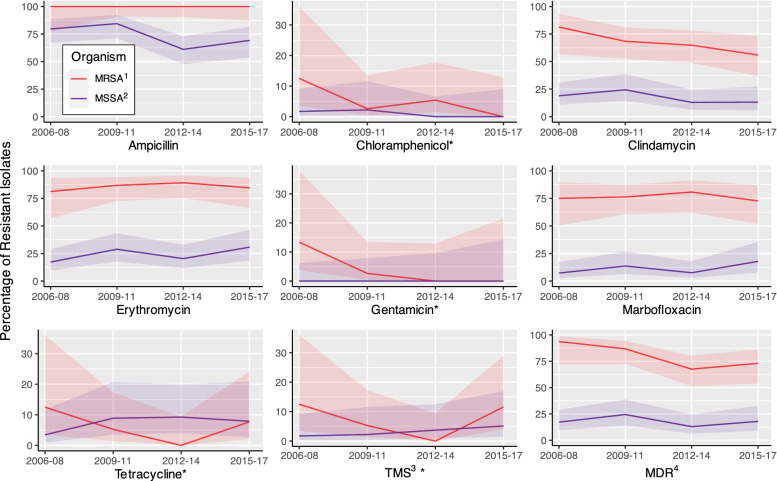
Percentage and 95% confidence intervals of antimicrobial and multidrug resistance among methicillin-resistant and methicillin-susceptible *S. aureus* isolated from canine specimens submitted to a veterinary diagnostic laboratory in Tennessee, USA (2006–2017). ^1^Methicillin-resistant *S. aureus*, ^2^Methicillin-susceptible *S. aureus*, ^3^Trimethoprim/sulfamethoxazole, ﻿^4^Multidrug resistance. *Indicates that upper limit of y-axis has been reduced to improve visual comparison

A significantly (*p* < 0.0001) higher proportion of MRSA isolates were MDR compared to MSSA (Fig. 2). Similarly, significant differences between MRSA and MSSA were identified for ampicillin (*p* < 0.0001), clindamycin (*p* < 0.0001), erythromycin (*p* < 0.0001), gentamicin (*p* = 0.0475), and marbofloxacin (*p* < 0.0001). There were significant decreases in the proportion of MRSA isolates resistant to gentamicin (*p* = 0.0400) and the proportion of MSSA isolates resistant to ampicillin (*p* = 0.0485) during the study period. In addition, the percentage of MDR MRSA isolates decreased significantly (*p* = 0.0298), from 93.8 to 73.1%.

iii) *S. coagulans*

Ampicillin (40.9%) resistance among *S. coagulans* isolates was comparatively lower than that of *S. pseudintermedius* and *S. aureus* (Table 4). Resistance to gentamicin (36.2%) and marbofloxacin (37.7%) were relatively common among *S. coagulans* isolates, while resistance to TMS, clindamycin, erythromycin, and tetracycline were rarely observed, and none exhibited resistance to chloramphenicol. Among the subsets of isolates tested for susceptibility to amikacin (232 isolates) and rifampin (28 isolates), none exhibited resistance.

**Table 4:** Distribution of antimicrobial and multidrug resistance among *S. coagulans* isolated from canine specimens submitted to a veterinary diagnostic laboratory in Tennessee, USA (2006–2017)

Antimicrobial	2006–2008	2009–2011	2012–2014	2015–2017	Total
**Aminoglycosides**					
Gentamicin	35.5 (67/194)	45.6 (93/204)	33.1 (48/145)	26.3 (30/114)	36.2 (238/657)
**β-lactams**					
Ampicillin	40.1 (75/187)	44.1 (90/204)	41.5 (107/258)	37.6 (76/202)	40.9 (348/851)
Cefoxitin	21.7 (42/194)	26.1 (53/203)	20.6 (53/257)	18.7 (38/203)	21.7 (186/857)
Oxacillin	35.6 (69/194)	41.2 (84/204)	40.9 (105/257)	31.0 (63/203)	37.4 (321/858)
**Fluoroquinolones**					
Marbofloxacin	30.9 (59/191)	44 (88/200)	41.1 (106/258)	33.3 (51/153)	37.7 (304/802)
**Folate inhibitors**					
TMS^a^	0.52 (1/194)	0.49 (1/204)	2.33 (6/257)	0.98 (2/204)	1.2 (10/859)
**Lincosamides**					
Clindamycin	4.6 (9/194)	3.4 (7/204)	2.3 (6/256)	3.4 (7/204)	3.4 (29/858)
**Macrolides**					
Erythromycin	4.1 (8/194)	2.9 (6/204)	2.7 (7/258)	2.9 (6/204)	3.1 (27/860)
**Phenicols**					
Chloramphenicol	0 (0/194)	0 (0/204)	0 (0/257)	0 (0/204)	0 (0/859)
**Tetracyclines**					
Tetracycline	1.6 (3/194)	2.0 (4/204)	3.1 (8/258)	3.0 (6/203)	2.4 (21/859)
**AMR**^b^	56.7 (110/194)	64.2 (131/204)	57.0 (147/258)	50.0 (102/204)	57.0 (490/860)
**MDR**^c^	21.7 (42/194)	30.9 (63/204)	16.7 (43/258)	11.8 (24/204)	20.0 (172/860)**
**MRSC**^d^	25.2 (42/167)	35.2 (70/199)	39.2 (100/255)	25.8 (49/190)	32.2 (261/811)

^a^Trimethoprim/sulfamethoxazole

^b^Antimicrobial resistance

^c^Multidrug resistance

^d^Methicillin-resistant *S. coagulans*, based on oxacillin disk diffusion testing confirmed with positive *mecA* PCR

*p < 0.05, **p < 0.01, ***p < 0.0001 for Cochran-Armitage trend test

The percentages of AMR (57%) and MDR (20%) *S. coagulans* isolates were much lower than those for *S. pseudintermedius* and *S. aureus*. Furthermore, MDR showed a significant (*p* = 0.0004) decreasing temporal trend, with the percentage of resistant isolates changing from 21.7% at the beginning of the study period to 11.8% at the end. On the other hand, overall methicillin resistance was similar to *S. pseudintermedius* and *S. aureus* (32.2%), but did not exhibit any significant temporal trends.

iv) *S. schleiferi*

Similar to findings for *S. coagulans, S. schleiferi* isolates also exhibited a lower level of resistance to ampicillin (56.5%) than *S. pseudintermedius* and *S. aureus* (Table 5)*.* However, *S. schleiferi* isolates had the highest level of methicillin resistance of all species in this study (56.9%). Approximately half of the *S. schleiferi* isolates showed resistance to gentamicin (45.5%) and marbofloxacin (48%), while resistance to other drugs, including TMS (2.5%), clindamycin (8.6%), erythromycin (8.6%), and tetracycline (6.8%) was much less common. However, resistance to tetracycline did increase significantly during the course of the study period (*p* = 0.0217). Among the subsets of isolates tested for susceptibility to amikacin (56 isolates) and rifampin (7 isolates), resistance to amikacin was observed in a single isolate, and none were resistant to rifampin.

**Table 5:** Distribution of antimicrobial and multidrug resistance among *S. schleiferi* isolated from canine specimens submitted to a veterinary diagnostic laboratory in Tennessee, USA (2006–2017)

Antimicrobial	2006–2008	2009–2011	2012–2014	2015–2017	Total
**Aminoglycosides**					
Gentamicin	41.03 (16/39)	48.3 (14/29)	50.0 (11/22)	45.5 (10/22)	45.5 (51/112)
**β-lactams**					
Ampicillin	60.5 (23/38)	55.2 (16/29)	45.0 (18/40)	63.0 (34/54)	56.5 (91/161)
Cefoxitin	20.5 (8/39)	17.2 (5/29)	32.5 (13/40)	30.2 (16/53)	26.1 (42/161)
Oxacillin	66.7 (26/39)	65.5 (19/29)	52.5 (21/40)	57.4 (31/54)	59.9 (97/162)
**Fluoroquinolones**					
Marbofloxacin	39.5 (15/38)	51.7 (15/29)	51.3 (20/39)	50 (23/46)	48.0 (73/152)
**Folate inhibitors**					
TMS^a^	0 (0/39)	3.5 (1/29)	2.5 (1/40)	3.7 (2/54)	2.5 (4/162)
**Lincosamides**					
Clindamycin	10.3 (4/39)	6.9 (2/29)	0 (0/40)	14.8 (8/54)	8.6 (14/162)
**Macrolides**					
Erythromycin	10.3 (4/39)	6.9 (2/29)	0 (0/40)	14.8 (8/54)	8.6 (14/162)
**Phenicols**					
Chloramphenicol	0 (0/39)	3.5 (1/29)	0 (0/40)	0 (0/54)	0.6 (1/162)
**Tetracyclines**					
Tetracycline	0 (0/39)	6.9 (2/29)	5 (2/40)	13.0 (7/54)	6.8 (11/162)*
**AMR**^b^	84.6 (33/39)	82.8 (24/29)	72.5 (29/40)	72.2 (39/54)	77.2 (125/162)
**MDR**^c^	28.2 (11/39)	34.5 (10/29)	20.0 (8/40)	27.8 (15/54)	27.2 (44/162)
**MRSS**^d^	61.8 (21/34)	64.3 (18/28)	48.7 (19/39)	55.8 (29/52)	56.9 (87/153)

^a^Trimethoprim/sulfamethoxazole

^b^Antimicrobial resistance

^c^Multidrug resistance

^d^Methicillin-resistant *S. schleiferi*, based on oxacillin disk diffusion testing confirmed with positive *mecA* PCR

*p < 0.05, **p < 0.01, ***p < 0.0001 for Cochran-Armitage trend test

The majority of *S. schleiferi* isolates were resistant to at least one antimicrobial (77.2%), but MDR was again comparatively lower than the other *Staphylococcus* species (27.2%). Methicillin resistance fluctuated but did not exhibit any significant temporal trends.

### Methicillin resistance and mecA gene detection

- *S. pseudintermedius*

A total of 97.4% of the cefoxitin-resistant *S. pseudintermedius* isolates (based on disk diffusion test) were positive for the *mecA* gene (based on PCR assay), while only 62.1% of cefoxitin-susceptible isolates were negative for *mecA* (Table 6). Overall, there was 74.9% agreement between cefoxitin disk diffusion testing and *mecA* PCR assay. The kappa test of agreement indicated moderate agreement (kappa = 0.522; *p* < 0.0001) between the two methods. In contrast, oxacillin disk diffusion test had almost perfect agreement with *mecA* PCR (95.4% agreement; κ = 0.904; *p* < 0.0001).

b) *S. aureus*

**Table 6:** Comparison of the results of *mecA* PCR and cefoxitin/oxacillin resistance among *Staphylococcus* spp. isolated from canine specimens submitted to a veterinary diagnostic laboratory in Tennessee, USA (2006–2017)

Organism	Drug	Interpretation	***mecA*** positive		***mecA*** negative		Percent	κ^a^	95% CI^b^		***p***-value
			Percent	Number	Percent	Number	agreement				
*S. pseudintermedius*	Cefoxitin	Resistant	97.4	1160/1191	2.6	31/1191	74.9%	0.522	0.497	0.548	< 0.0001
		Susceptible	37.9	797/2104	62.1	1307/2104					
	Oxacillin	Resistant	95.4	1908/2001	4.7	93/2001	95.4%	0.904	0.889	0.919	< 0.0001
		Susceptible	4.6	60/1311	95.4	1251/1311					
*S. aureus*	Cefoxitin	Resistant	96.7	117/121	3.3	4/121	95.3%	0.834	0.714	0.953	< 0.0001
		Susceptible	12.0	3/25	88.0	22/25					
	Oxacillin	Resistant	95.9	117/122	4.1	5/122	94.0%	0.787	0.653	0.920	< 0.0001
		Susceptible	16.0	4/25	84.0	21/25					
*S. coagulans*	Cefoxitin	Resistant	94.4	151/160	5.6	9/160	76.3%	0.525	0.460	0.590	< 0.0001
		Susceptible	31.1	121/389	68.9	268/389					
	Oxacillin	Resistant	95.3	261/274	4.7	13/274	95.6%	0.913	0.878	0.947	< 0.0001
		Susceptible	4.0	11/275	96.0	264/275					
*S. schleiferi*	Cefoxitin	Resistant	97.4	37/38	2.6	1/38	59.1%	0.291	0.182	0.400	< 0.0001
		Susceptible	57.3	51/89	42.7	38/89					
	Oxacillin	Resistant	98.9	87/88	1.1	1/88	97.7%	0.945	0.884	1.000	< 0.0001
		Susceptible	5.0	2/40	95.0	38/40					

^a^Kappa statistic for test of agreement

^b^Confidence interval

Compared to *S. pseudintermedius*, *S. aureus* isolates had higher overall agreement (almost perfect) between the results of cefoxitin disk diffusion and *mecA* PCR (95.3% agreement; κ = 0.834; *p* < 0.0001). There was also substantial agreement between oxacillin disk diffusion and *mecA* PCR assay (94.0% agreement; κ = 0.787; *p* < 0.0001).

iii) *S. coagulans* and *S. schleiferi*

The findings for *S. coagulans* and *S. schleiferi* isolates were similar to those for *S. pseudintermedius*, with oxacillin disk diffusion being more consistent with the results of *mecA* PCR assay than cefoxitin disk diffusion. Cefoxitin disk diffusion tests had moderate agreement with *mecA* PCR for *S. coagulans* isolates (76.3% agreement; κ = 0.525; *p* < 0.0001), and fair agreement for *S. schleiferi* isolates (59.1% agreement; κ = 0.291; *p* < 0.0001). In contrast, the agreement between oxacillin disk diffusion tests and *mecA* PCR was almost perfect for both *S. coagulans* (95.6% agreement; κ = 0.913; *p* < 0.0001) and *S. schleiferi* (97.7% agreement; κ = 0.945; *p* < 0.0001).

## Discussion

This study assessed patterns of antimicrobial, multidrug and methicillin resistance among *Staphylococcus* isolates from canine clinical specimens submitted to the UTCVM Bacteriology Laboratory between 2006 and 2017. The majority of these staphylococci were isolated from skin, ear and mucosal specimens, reflecting their importance as causative agents of pyoderma and otitis [3–6], which are frequently encountered in clinical practice. Surveillance of antimicrobial susceptibility patterns among canine *Staphylococcus* isolates is therefore valuable for guiding evidence-based treatment decisions, and useful for identifying trends that may reflect selection pressure.

### Patterns of antimicrobial and multidrug resistance

- *S. pseudintermedius*

The majority of *S. pseudintermedius* isolates were resistant to ampicillin (84.3%) and therefore most were classified as AMR, consistent with previous reports [28, 47, 61, 62]. Indeed, resistance to penicillinase-labile penicillins has become widespread over the past several decades [63], limiting their usefulness for the treatment of staphylococcal infections in dogs.

Relatively high levels of MRSP were observed throughout the study period (30.8% overall), with a mild but statistically significant temporal increase. This follows a steep increase in *mecA*-mediated resistance among isolates submitted to the UTCVM bacteriology laboratory between 2001 and 2007 [55]. These findings are not unprecedented, as *mecA-*mediated resistance has emerged among *S. pseudintermedius* in North America and Europe [64, 65], and MRSP has been detected around the world [66–71]. However, the level of methicillin resistance among isolates from canine specimens in the current study was higher than previously observed in other locations within the U.S., such as Kentucky [14] and Michigan [72]. These regional differences imply that geographically relevant AMR surveillance provides valuable information for veterinary practitioners.

In addition to the substantial level of methicillin resistance, almost half of the *S. pseudintermedius* isolates in the current study were MDR. Though a temporal decline in the proportion of MDR MRSP isolates was observed, the vast majority of MRSP (90.5%) were MDR. A limited number of antimicrobials are available to effectively treat MDR and MRSP infections; these isolates are often resistant to drug classes used in veterinary medicine such as tetracyclines, fluoroquinolones, macrolides, trimethoprim-sulfonamides, and lincosamides [73]. Considerable levels of resistance to the above agents (exceeding 75%) were observed among MRSP isolates in the current study.

As a result of such limitations in available treatment options, infections with MDR and/or MRSP isolates may necessitate the use of antimicrobials such as rifampin or chloramphenicol, despite the potential for adverse effects [73–76]. Resistance to rifampin was only assessed among a subset of MDR isolates in the current study, and was infrequently observed. On the other hand, it is concerning that a statistically significant increase in resistance to chloramphenicol occurred among both MRSP and MSSP isolates during the study period, with the highest levels among MRSP (from 4.0 to 12.0%). This finding is particularly concerning given its utility for the treatment of MRSP infections. A similar but accelerated trend was reported among canine isolates collected at the Texas A&M Veterinary Teaching Hospital, likely due to selection pressure from the increased use of chloramphenicol in the face of MDR/MRSP infections [77]. However, while that study also reported an increase in resistance to amikacin [77], another potential treatment for MDR or MRSP infections [73], resistance to amikacin was rare among MDR and cutaneous isolates in the current study.

In addition to the clinical challenge associated with the treatment of MDR and MRSP infections in veterinary patients, the zoonotic potential of *S. pseudintermedius* raises concerns from a public health perspective. Human infections with *S. pseudintermedius* are typically associated with companion animal contact [43, 78–82], and have included MRSP infections [83, 84]. Given the potential for rapid selection of resistant organisms, ongoing surveillance is warranted to ensure prompt recognition of clinically relevant patterns of AMR and MDR. Indeed, while higher levels of resistance to non-β-lactam antibiotics among MRSP compared to MSSP are expected [63], the temporal increase in MDR MSSP isolates observed in the current study was an interesting, albeit concerning finding. This trend appears to have been driven by rising levels of resistance to fluoroquinolones, folate inhibitors, lincosamides, and macrolides, all of which exhibited decreases for MRSP isolates.

b) *S. aureus*

*S. aureus* was less commonly isolated in comparison to *S. pseudintermedius* in this study, as dogs are not the primary reservoir for this organism [85]. In the current study, 37.4% of *S. aureus* isolates were methicillin-resistant, and MRSA increased substantially during the study period (from 21.6 to 40%). Varying levels of methicillin resistance have been reported among canine clinical *S. aureus* isolates in other recent studies, ranging from 12.8% in an Australian study [86], to 62.7% of isolates from wound infections in a German study [87].

*S. aureus* strains in companion animals tend to mirror those circulating in humans [87, 88], and canine colonization and infections with *S. aureus* often result from reverse zoonosis [45, 89–91]. However, pets may serve as reservoirs once exposed, and MRSA carriage in humans may be difficult to eliminate if household dogs harboring the organism are not treated [45, 90]. The susceptibility patterns of *S. aureus* differed somewhat from *S. pseudintermedius*, which may reflect antimicrobial use patterns, particularly if humans are a major source of *S. aureus* colonization in this population of dogs. For example, resistance to gentamicin, tetracycline, and TMS were much less common among *S. aureus*, and a temporal trend was not observed for chloramphenicol. Furthermore, the proportion of MDR isolates did not increase among MSSA. However, a more rapid increase in methicillin resistance was observed among *S. aureus* isolates compared to *S. pseudintermedius*. Further investigation is warranted to understand the epidemiology of *S. aureus* in this patient population and identify factors which may have contributed to the observed patterns, including the pronounced increase in MRSA.

iii) *S. coagulans* and *S. schleiferi*

While *S. coagulans* and *S. schleiferi* (formerly *S. schleiferi* subsp. *coagulans* and *S. schleiferi* subsp. *schleiferi* [15]) have received increased attention for their roles as human and animal pathogens in recent years, there are fewer published reports of AMR patterns for these organisms in comparison to *S. pseudintermedius* and *S. aureus.* The percentage of methicillin resistant isolates was the most notable difference between *S. coagulans* and *S. schleiferi* (32.2 and 56.9%, respectively), a finding which has previously been reported [19]. In fact, *S. schleiferi* isolates had the highest proportion of methicillin resistant isolates in the current study, consistent with previous reports [19, 92]. In addition to being isolated from healthy dogs and first-time infections, *S. coagulans* and *S. schleiferi* may be particularly important in recurrent infections such as otitis and pyoderma [17, 18, 93–95]. Prior exposure to β-lactam antibiotics, commonly used for empirical treatment of skin infections, is a reported risk factor for oxacillin (methicillin) resistance among *S. coagulans* and *S. schleiferi* isolates from canine specimens [19].

Despite the considerable proportion of methicillin-resistant *S. schleiferi* isolates, several other antimicrobial classes appear to remain as potential treatment options. In general, similar patterns of resistance to non-β-lactam antibiotics were observed between *S. coagulans* and *S. schleiferi*. Resistance to TMS, clindamycin, erythromycin, chloramphenicol and tetracycline were relatively uncommon, while higher levels of resistance to gentamicin and marbofloxacin were observed, in agreement with the findings of Kunder and colleagues [92]. While the percentages of MDR *S. coagulans* and *S. schleiferi* (20 and 27.2%) were much lower than the other *Staphylococcus* spp., they were also comparable with the findings of the above study (21%) [92]. Encouragingly, a significant temporal decline in MDR occurred among *S. coagulans*, despite the finding that few such trends were observed for individual agents or drug classes. Further research is warranted to investigate how this finding relates to patterns of antimicrobial use in the region, and to elucidate the role of *S. coagulans* and *S. schleiferi* as pathogens and commensal organisms of dogs.

### Methicillin resistance and mecA gene detection

The findings of the current study are consistent with previous reports, which have identified oxacillin disk diffusion as a superior method for detecting *mecA-*mediated methicillin resistance in *S. pseudintermedius, S. coagulans,* and *S. schleiferi* when compared to cefoxitin [55, 56, 96, 97]. This is reflected in the current CLSI standards [98]. While there was almost perfect agreement between oxacillin disk diffusion and PCR, a small proportion of oxacillin-susceptible *S. pseudintermedius, S. coagulans* and *S. schleiferi* isolates were *mecA-*positive. Detection of the *mecA* gene in oxacillin-susceptible isolates may reflect the effects of regulatory elements on *mecA* gene expression and subsequent PBP2a production [31, 67, 99]. On the other hand, a small percentage (< 5%) of oxacillin-resistant *S. pseudintermedius, S. coagulans* and *S. schleiferi* isolates were *mecA-*negative, in contrast with the findings of several studies that reported *mecA* gene detection in all phenotypically resistant isolates [31, 55, 96]. One study, however, identified a single canine *S. pseudintermedius* isolate speculated to have an alternate, non-*mecA*-mediated mechanism of oxacillin resistance. The isolate in question had a small zone size and low minimum inhibitory concentration (MIC) for oxacillin, but neither the *mecA* nor *mecC* genes were detected [97].

While MIC values may be useful when interpreting discordant results, these were not available for most isolates in the current study. Among the *S. pseudintermedius* isolates in question, 19 had recorded MIC values; 13 had zone sizes at or near the interpretive breakpoint but would be considered susceptible based on their MIC (≤0.25 μg/mL) [52]. For isolates that did not have borderline zone sizes and/or MIC values, the possibility of an alternate mechanism of oxacillin resistance cannot be excluded based on the information available. Nonetheless, results of the current study support the usefulness of oxacillin disk diffusion for detecting *mecA-*mediated resistance in *S. pseudintermedius, S. coagulans,* and *S. schleiferi*.

For *S. aureus*, cefoxitin was superior to oxacillin disk diffusion for detecting *mecA-*mediated resistance, consistent with CLSI recommendations [52, 98]. While there was almost perfect agreement between the two tests, discordant results were obtained for seven *S. aureus* isolates. In order to facilitate comparison between studies, CLSI-recommended interpretive breakpoints in place at the time of sample submission were used in the current study. The recommended cutoff values for cefoxitin changed during the study period, from ≤19 mm (resistant), ≥20 mm (susceptible) to ≤21 mm (resistant), ≥22 mm (susceptible) [49, 50]. Under current CLSI standards, one of the three *mecA-*positive, cefoxitin-susceptible isolates would have been re-classified as cefoxitin-resistant, resulting in agreement between the two tests.

Further investigation to determine the mechanism of phenotypic methicillin resistance for the *mecA*-negative *Staphylococcus* spp. isolates was beyond the scope of the present study. However, the identification of this pattern among several *Staphylococcus* spp. isolates in this study suggests that PCR assay for both the *mecA* and *mecC* genes may be warranted in future research.

### Study strengths and limitations

The identification of antimicrobial resistance patterns and trends using a large sample of canine patients in this study are useful for informing evidence-based treatment decisions. In addition, clinical specimens were obtained from submissions by referring veterinarians in addition to hospital patients, representing a broad range of patients from both primary practice and specialty services. Antimicrobial susceptibility data were reported at the level of individual drugs, allowing for a detailed assessment of these patterns.

However, this study is not without limitations. For instance, while *Staphylococcus intermedius* group (SIG) species cannot be reliably distinguished by biochemical identification processes, virtually all canine SIG isolates are *S. pseudintermedius*; therefore, as recommended, SIG isolates were reported as *S. pseudintermedius* [100, 101]. *S. coagulans* and *S. schleiferi* were differentiated based upon tube coagulase testing, which may be inconsistent with genotypic testing [102], and therefore some of these isolates may have been misclassified. In addition, patient medical history, including current or prior antimicrobial therapy, was not available, precluding our ability to identify drivers of the observed patterns of AMR and MDR. Finally, the use of specimens submitted for culture may present some selection bias, as these are unlikely to be from first-time infections, and may have been submitted due to therapeutic failure. However, the majority of specimens were from submissions by referring veterinarians, and samples were not solely obtained from patients of specialty services, resulting in a broader picture of AMR patterns across a variety of clinical settings. Despite the above limitations, the findings of the current study provide valuable insights to patterns and trends of AMR in the region, and are useful for guiding both clinical decisions and directions for future research.

## Conclusion

There is evidence of substantial levels of MDR among *S. pseudintermedius* and *S. aureus* isolates from canine specimens, and increasing temporal trends in MRSP and MRSA. Unfortunately, this suggests that few agents remain broadly effective against these organisms. Furthermore, the emergence of chloramphenicol resistance observed in the current study is concerning. Future studies will investigate potential drivers of the observed antimicrobial susceptibility patterns. The observed levels of MDR and methicillin-resistance highlight the importance of antibiograms to guide treatment decisions. The temporal increase in canine methicillin-resistant staphylococci observed in this study has practical implications for human health. These findings highlight the need for continued surveillance of antimicrobial resistance patterns among staphylococci, and the importance of judicious antimicrobial use practices.

## Data Availability

The dataset used and/or analyzed during the current study is available from the corresponding author on reasonable request.

## Abbreviations

AKC: American Kennel Club
AMR: Antimicrobial Resistance
COE: Centers of Excellence
CLSI: Clinical and Laboratory Standards Institute
IQR: interquartile range
MDR: Multidrug Resistance
MIC: minimum inhibitory concentration
MRS: Methicillin resistant staphylococci
MRSA: methicillin-resistant *Staphylococcus aureus*
MRSC: methicillin-resistant *Staphylococcus coagulans*
MRSP: methicillin-resistant *Staphylococcus pseudintermedius*
MRSS: methicillin-resistant *Staphylococcus schleiferi*
PBP: penicillin binding protein
PCR: Polymerase Chain Reaction
SIG: *Staphylococcus intermedius* group
TMS: trimethoprim/sulfamethoxazole
UTCVM: University of Tennessee College of Veterinary Medicine
UTVTH: University of Tennessee Veterinary Teaching Hospital
